# *Fucus* *vesiculosus* Polysaccharide-Based Solid Dispersions Enhance Aqueous Dispersion and Gut Microbial Biotransformation of Ellagic Acid

**DOI:** 10.3390/foods15152587

**Published:** 2026-07-23

**Authors:** Rui-Bo Jia, Dapei Ou, Fenghua Liang, Zhao-Rong Li, Jinsong Wang, Chunxia Zhou, Pengzhi Hong

**Affiliations:** 1 College of Food Science and Technology, Guangdong Ocean University, Guangdong Provincial Key Laboratory of Aquatic Product Processing and Safety, Guangdong Province Engineering Laboratory for Marine Biological Products, Guangdong Provincial Engineering Technology Research Center of Seafood, Guangdong Provincial Engineering Technology Research Center of Prefabricated Seafood Processing and Quality Control, Zhanjiang 524088, China; jiaruibo@hotmail.com (R.-B.J.);; 2 School of Food Science and Engineering, South China University of Technology, Guangzhou 510640, China; 3 Institute of Food Nutrition and Health, Open Project of Hubei Key Laboratory of Drug Synthesis and Optimization, Jingchu University of Technology, Jingmen 448000, China

**Keywords:** ellagic acid, *Fucus vesiculosus* polysaccharide, solid dispersion, urolithins, gut microbiota

## Abstract

Ellagic acid (EA) is a food-derived polyphenol with multiple biological activities, but its poor water solubility and aggregation in aqueous systems limit its accessibility to gut microorganisms. In this study, *Fucus vesiculosus* polysaccharide (FVP) was used as a natural carrier to prepare EA/FVP solid dispersions (SDs) with different mass ratios by solvent-assisted evaporation. Compared with pure EA, the SDs markedly improved the apparent solubility and aqueous dispersion stability of EA. The highest apparent solubility was observed for the EA/FVP 3/1 formulation (0.75 ± 0.03 mg/mL), which was approximately 6.25-fold higher than that of pure EA (0.12 ± 0.01 mg/mL). The improved dispersion behavior was associated with increased apparent viscosity, reduced aggregation, decreased crystallinity and possible non-covalent interactions between EA and the polysaccharide matrix. *In vitro* fecal fermentation showed that SDs enhanced the production of urolithins, including urolithin M5, urolithin M6, urolithin C, iso-urolithin A and urolithin B, without generating new metabolite types. The EA/FVP 1/3 formulation showed the strongest promotion of urolithin production, with iso-urolithin A reaching 2.36 ± 0.28 μM. 16S rRNA sequencing showed that the enhanced urolithin production was accompanied by shifts in the fecal microbial community. Several bacterial genera positively correlated with urolithin biosynthesis, including *Bacteroides*_H, *Negativicoccus*, *Parabacteroides*_B, *Unclassified_Eggerthellaceae* and *Lactococcus*_A, were significantly enriched in the SDs groups. These findings suggest that FVP-based SDs may serve as a potential strategy for improving the apparent solubility, aqueous dispersion and microbial biotransformation of EA.

## 1. Introduction

Ellagic acid (EA) is a food-derived polyphenolic compound with a molecular formula of C_14_H_6_O_8_ [1]. EA possesses excellent biological activities, including antioxidant, anti-inflammatory, and anti-cancer effects and regulation of intestinal flora [2]. However, EA has extremely poor solubility, which significantly limits the exertion of its health-promoting functions. Urolithins are the intestinal microbial metabolites of EA. Compared to the parent compound EA, urolithins not only exhibit favorable similar biological functions, but also show higher bioavailability [3]. Therefore, urolithins are generally recognized as the potential contributors to health benefits of foods rich in EA.

Microbial biotransformation is widely used in the pharmaceutical industry, serving as an effective approach to obtain metabolic compounds. For example, Sary et al. screened 17 microbial strains to explore microbial biotransformation for generating novel carotol derivatives with potential pharmacological value, and found that *Absidia coerulea* ATCC 6647 was the most effective biocatalyst to produce three new metabolites: CM1, CM2, and CM3, in yields of 30%, 9.96%, and 3.28%, respectively [4]. Manhas et al. used *Talaromyces purpurogenus* strain MRS-F13 to biotransform (-)-verbenone into (-)-10-hydroxyverbenone, and the resulting product exhibited enhanced antioxidant activity [5]. In recent years, a growing number of studies have focused on identifying the bacterial strains responsible for converting EA to urolithins [6]. He et al. performed an in vitro fermentation of EA and the results revealed that *Bifidobacterium longum*, *Bifidobacterium adolescentis*, and *Bifidobacterium bifidum* might be involved in the conversion of EA to urolithin A [7]. Zhang et al. employed *Caenorhabditis elegans* as a model organism to screen edible urolithin A-producing strains, and the results showed that the *Lactobacillus plantarum* strains CCFM1286, CCFM1290, and CCFM1291 could convert ellagitannin to produce urolithin A [8]. However, when incubating EA with the aforementioned strains, the urolithin conversion ratio remains relatively low. Consequently, the microbial-based acquisition of urolithins still faces considerable challenges at present.

Substrate solubility is a fundamental factor affecting its metabolic process. For instance, in the progesterone biotransformation by *Mucor racemosus*, the addition of Tween 80 significantly improved the progesterone biotransformation efficiency, reaching 81.56 ± 2.29% [9]. García-Villalba et al. observed that EA dissolved in 1% dimethyl sulfoxide (DMSO) in the culture medium was more effectively metabolized compared to the group without DMSO. Additionally, the presence of DMSO facilitated the formation of other metabolic intermediates, indicating that the EA biotransformation process was influenced by the nature of its solubility [10]. These studies demonstrated that organic solvent addition can enhance the solubility of insoluble substrate and improve their accessibility to microorganisms, thereby promoting the biotransformation process. However, the excessive use of organic solvents may inhibit microbial growth and accelerate deactivation of the biocatalyst during the bioconversion. Therefore, it is necessary to develop safer and more effective strategies to promote microbial biotransformation.

Solid dispersion is an effective strategy for improving the dissolution behavior of poorly soluble compounds. It refers to a system in which poorly soluble substances are dispersed in a carrier in a molecular, amorphous or metastable microcrystalline state, thereby enhancing their apparent solubility and dissolution rate [11]. For example, Song et al. prepared a lutein co-amorphous formulation with enhanced solubility and dissolution [12]. Nyamba et al. conducted a preformulation study for the development of EA-based solid dispersions using hot-melt extrusion [13]. *Fucus vesiculosus* is a brown alga widely distributed along the western coast of France and the Bohai Sea of China, and its polysaccharides are commonly used as carriers to construct delivery systems for hydrophobic active substances [14]. Previous studies showed that fucoidan from *Fucus vesiculosus* could serve as materials for nanoparticle production and cell compatibility [14,15]. Zhang et al. used fucoidan-stabilized zein nanoparticles to encapsulate curcumin and evaluate its in vitro release performance [16]. In addition, food-derived EA-*Undaria pinnatifida* polysaccharide solid dispersions were prepared in which great improvements in the solubility, dispersity and biotransformation of EA were observed [17]. These studies show that fucoidan-based solid dispersions could improve the dissolution or dispersion of poorly soluble compounds. Therefore, in the current study, we hypothesized that FVP could improve EA dispersion and increase the fraction of EA accessible to fecal microbiota by reducing the EA crystallization and aggregation through non-covalent interactions. To test this hypothesis, EA/FVP solid dispersions with different mass ratios were prepared by solvent evaporation. Their physicochemical properties were characterized by apparent solubility, suspension stability, particle size, PDI, UV–vis spectroscopy, FT-IR, XRD, DSC, SEM and steady-shear rheology; their effects on EA biotransformation and microbiota composition were then evaluated using an in vitro fecal fermentation model and 16S rRNA gene sequencing.

## 2. Materials and Methods

### 2.1. Materials and Reagents

*Fucus vesiculosus* was purchased from Qingdao Fuxingxiang Import &Export Co., Ltd. (Qingdao, Shandong, China). The materials were thoroughly washed, dried (45 °C), and then placed in a sample chamber with constant temperature and humidity for further use.

EA (purity ≥ 90%) was purchased from Xi’an Mixianer Biotechnology Co., Ltd. (Xi’an, Shaanxi, China). Standards of EA (purity ≥ 96%), urolithin B (purity ≥ 98%), urolithin C (purity ≥ 95%), and urolithin M5 (purity ≥ 95%) were purchased from Shanghai Yuanye Bio-technology Co., Ltd. (Shanghai, China). Urolithin M6 standard (purity ≥ 98%) and 1-methyl-2-pyrrolidinone (NMP, purity ≥ 98%) were obtained from Weikeqi Bio-technology Co., Ltd. (Chengdu, Sichuan, China) and Shanghai Macklin Biochemical Technology Co., Ltd. (Shanghai, China), respectively. LC-MS grade acetonitrile (ACN), formic acid, and ultrapure water were obtained from Fisher Scientific (Loughborough, UK), Aladdin (Shanghai, China), and a Milli-Q system (Millipore, Bedford, MA, USA), respectively.

### 2.2. Preparation of Fucus vesiculosus Polysaccharide (FVP)

In our previous published studies, the extraction, separation, characterization and biological activities of FVP were systematically investigated [18,19]. Briefly, the extraction procedure was as follows: *Fucus vesiculosus* (100 g) was immersed in 1500 mL deionized water for 1 h and extracted at 90 °C for 2 h with stirring. After cooling, the mixture was centrifuged (8000× *g*, 20 min, 25 °C) and filtered. The supernatant was concentrated, and then precipitated with four volumes of absolute ethanol at 4 °C for 12 h. The precipitates were redissolved and ultrafiltered using a membrane with 5 kDa cut-off to remove low-molecular-weight components. Subsequently, the solution was concentrated. The polysaccharides were lyophilized.

The total sugar contents were determined by the phenol sulfuric acid method with L-fucose as the standard, the protein content was measured using the Kjeldahl nitrogen method, total phenol content was determined by the Folin–Ciocalteu method with gallic acid as the standard, uronic acid content was analyzed by the sulfuric acid-carbazole method using glucuronic acid to make the standard curve, and sulfate content was measured by the turbidimetric method. Monosaccharide composition was analyzed using a Waters e2695 HPLC system (Waters Corporation, Milford, MA, USA) equipped with a UV detector (Waters Corporation, Milford, MA, USA) after acid hydrolysis and PMP derivatization [18]. The molecular weight was determined by high-performance size-exclusion chromatography coupled online with multi-angle laser light scattering and refractive-index detectors (HPSEC-MALLS-RI) [18]. The results showed that the total sugar, protein, uronic acid and sulfate content of FVP were 77.90 ± 0.74%, 1.67 ± 0.20%, 21.01 ± 0.63% and 9.72 ± 0.31%, respectively. FVP was found to consist of fucose (45.84%), xylose (23.22%), mannose (13.28%), glucuronic acid (8.68%), glucose (4.93%), and galactose (4.06%). The average molecular weight of FVP was measured to be 281.9 kDa. The relatively high fucose proportion, together with uronic acid and sulfate groups, indicates the fucose-rich acidic sulfated polysaccharide characteristics of FVP.

### 2.3. Preparation of EA/FVP SDs

The EA/FVP SDs were prepared by the solvent evaporation method. Briefly, EA was dissolved in 60% ethanol at 50 °C at 0.1 wt % concentration, while FVP (the carrier) was dissolved in deionized water at 0.1 wt % concentration. When totally dissolved, the corresponding FVP solution was slowly added to the EA solution while stirring. A series of mass ratios (EA/FVP, *w*/*w*) including 5/1, 3/1, 1/1, 1/3, and 1/5 were used to prepare EA/FVP SDs. After 24 h of agitation, the solvent was removed by rotary evaporation at 45 °C under reduced pressure of approximately −0.1 MPa, and the EA and FVP were precipitated together. The precipitates on the inner wall of the bottle were scraped off and placed in the oven to dry for 12 h. The dried samples were ground in a mortar to obtain light-yellow powders, which were named according to their EA/FVP mass ratios. Equal masses of EA and FVP were weighed and mixed using a vortex mixer (Thermo Fisher Scientific, Waltham, MA, USA) as control, and the physical mixture was called PM 1/1. All samples were stored in a desiccator for further use.

### 2.4. High-Performance Liquid Chromatography (HPLC) Analysis of EA and Urolithins

The amount of EA and urolithins was determined using a U3000 HPLC system (Thermo Fisher Scientific, Waltham, MA, USA) equipped with a UV detector. Chromatographic separation was performed on a Thermo Acclaim C18 column (Thermo Fisher Scientific, Waltham, MA, USA) (4.6 × 250 mm, 5 μm) at 30 °C. The mobile phase consisted of 0.1% formic acid in water (A) and acetonitrile (B), with a flow rate of 1.0 mL/min, an injection volume of 10 μL and a detection wavelength of 254 nm. The gradient program was as follows: 0–5 min, 88–65% A; 6–10 min, 65% A; 10–14 min, 65–5% A; 14–16 min, 5% A; 16–18 min, 5–88% A; and 18–20 min, 88% A. For fermentation samples, 5 mL fermentation broth was extracted with 10 mL ethyl acetate containing 1.5% formic acid (*v*/*v*), vortexed for 1 min and centrifuged at 3500 rpm for 8 min. The organic phase was collected, evaporated to dryness and redissolved in NMP before HPLC analysis. EA and urolithins were identified by comparing retention times with authentic standards [17]. Calibration curves were constructed for EA and the detected urolithins, with correlation coefficients (R^2^) above 0.9989; all quantified samples were within the corresponding linear ranges.

### 2.5. Physicochemical Characterization of EA/FVP SDs

#### 2.5.1. Equilibrium Apparent Solubility

Samples were accurately weighed and added in a centrifuge tube with 5 mL of water to keep the EA concentration at 1 mg/mL. Then, the mixtures were dissolved in a shaking water bath at a speed of 100 r/min for 6 h to achieve dissolution equilibrium of samples, and left to stand for 30 min to precipitate the undissolved samples at the bottom. The apparent equilibrium solubility was measured by determining the EA contents in the supernatant. Next, 200 μL of supernatant was diluted and extracted 4 times with NMP. After fully vortexing, the solution was filtered through a 0.22 μm PTFE membrane filter and analyzed by HPLC. The HPLC method is described in Section 2.4.

#### 2.5.2. Turbidity Determination

To evaluate the turbidity of the samples, the optical density at 600 nm (OD_600_) was monitored utilizing a Readmax 1900 microplate reader (Shanghai Flash Spectrum Biotechnology Co., Ltd., Shanghai, China). First, all specimens were dissolved in deionized water to achieve a final concentration of 0.1% (*w*/*v*), followed by thorough homogenization via a vortex mixer. The absorbance values of the resulting supernatants were sequentially documented at designated intervals of 0, 15, 30, 60, 90, and 120 min. Then, after the mixture was allowed to stand for 24 h at ambient temperature, the OD_600_ of the supernatants was measured once more.

#### 2.5.3. Particle Size, PDI and Zeta Potential Determination

The particle size, PDI and zeta potential analysis of EA, FVP and SDs were characterized using a 90Plus PALS instrument (Brookhaven, Nashua, NH, USA). The samples were prepared in deionized water at a concentration of 0.02% (*w*/*v*). The resulting suspensions were transferred into the sample cell for measurement. The indexes were detected at 25 °C.

#### 2.5.4. UV–Vis Spectroscopy Analysis

Appropriate amounts of EA, FVP, PM 1/1 and EA/FVP SDs were dispersed in water, and UV–vis spectra were recorded from 200 to 600 nm at room temperature using a Readmax 1900 microplate reader (Shanghai Flash Spectrum Biotechnology Co., Ltd., China).

#### 2.5.5. Fourier Transform Infrared Spectrometer (FT-IR)

The FT-IR spectra of EA, FVP and EA/FVP SDs were acquired on a Bruker VERTEX 33 spectrometer (Bruker Corporation, Bremen, Germany) over a scanning range of 4000–400 cm^−1^. Each sample was individually ground with KBr powder and pressed into pellets according to the KBr disc method.

#### 2.5.6. X-Ray Diffraction (XRD) Analysis

To investigate the crystalline structure of EA, FVP, and EA/FVP SDs, X-ray diffraction analysis was performed using a SmartLab diffractometer system (Rigaku, Tokyo, Japan). The measurements were conducted at ambient temperature with a Cu-K*α* radiation source. The scanning range of 2*θ* was set from 5° to 40° at a constant scan speed of 2°/min. Subsequently, the crystallinity index (CI) of each sample was evaluated based on a previously reported method.

#### 2.5.7. Differential Scanning Calorimetry (DSC) Analysis

Thermal profiles of the samples (approximately 5 mg) were characterized using a DSC Q2000 instrument (TA Instruments, New Castle, DE, USA) under a nitrogen (N_2_) atmosphere with a flow rate of 50 mL/min. The specimens were heated from 50 °C to 400 °C at a constant heating rate of 10 °C/min.

#### 2.5.8. Rheological Properties

The steady-state shear rheological properties of EA, FVP and EA/FVP SDs were measured using a rheometer (HAAKE MARS III, Shanghai, China) equipped with a 35 mm titanium parallel-plate geometry at 25 °C. Before measurements, each sample was dispersed in deionized water at a concentration of 10 mg/mL. The apparent viscosity was recorded as a function of shear rate over the range of 0.1–100 s^−1^ to evaluate the flow behavior and dispersion stability of the samples.

#### 2.5.9. Scanning Electron Microscopy (SEM)

The SEM analysis of EA, FVP and EA/FVP SDs were carried out using a scanning electron microscope (S3400N, Hitachi Ltd., Tokyo, Japan) and sputtered with gold powder. The microstructure of samples was obtained at an accelerating voltage of 5 kV.

### 2.6. In Vitro Fermentation

Firstly, the basic anaerobic nutrient medium was prepared in the following proportions: 37.0 g/L brain-heart infusion (BHI), 0.7 g/L L-cysteine and 1.0 mL/L 1% resazurin solution. The experiment was divided into five groups: Control group (0.10 wt % EA added to basic medium), EA/FVP 3/1 group (0.13 wt % EA/FVP 3/1 added to basic medium), EA/FVP 1/1 group (0.20 wt % EA/FVP 1/1 added to basic medium), EA/FVP 1/3 group (0.40 wt % EA/FVP 1/3 added to basic medium), and PM 1/1 group (0.20 wt % PM 1/1 added to basic medium). The EA addition in the media was 0.1% wt among all groups. All media were prepared under anaerobic conditions with the atmosphere consisting of N_2_/H_2_/CO_2_ (80:10:10) at 37 °C and a volume of 9 mL was integrated into a glass anaerobic tube. The isolated media was autoclaved at 121 °C for 20 min, and cooled down to room temperature for further use.

The stool samples were collected from five healthy volunteers (three females and two males) who followed a regular diet and had not used antibiotics in the past 3 months or longer. The study was approved by the Ethics Committee of the College of Food Science and Technology, Guangdong Ocean University (Approval No. GDOU-202503), and written informed consent was obtained from all volunteers before sample collection. Equal amounts of stool samples were pooled to reduce inter-individual variability and to prepare a shared fecal inoculum for all treatment groups. The pooled sample was homogenized with sterile saline (stool:saline = 1:9, *w*/*v*) and filtered through sterile gauze under anaerobic conditions. One milliliter of fecal suspension was added to the sterilized medium described above. All groups were incubated anaerobically at 37 °C for 6 days, and each treatment was performed in triplicate technical replicates using the same pooled fecal inoculum.

### 2.7. 16S rRNA Amplicon Sequencing

Bacterial DNA from fermentation samples was extracted according to a previously reported method. The V3–V4 region of the bacterial 16S rRNA gene was amplified using primers 338F (5′-CCTACGGRRBGCASCAGKVRVGAAT-3′) and 806R (5′-GGACTACNVGGGTWTCTAATCC-3′). Paired-end sequencing was performed on an Illumina platform by Suzhou Biodeep Biomedical Technology Co., Ltd. (Suzhou, China). Raw reads were processed using QIIME2 (version 2019.4) with the DADA2 pipeline for quality control, and sequencing depth was evaluated based on the high-quality reads retained after filtering. Taxonomic assignment was performed against the Greengenes database (Release 13.8).

### 2.8. Statistical Analysis

Experimental data were analyzed using SPSS software (version 23.0). Significant differences among physicochemical and metabolite data were evaluated by one-way ANOVA followed by Duncan’s multiple range test. Data are presented as mean ± standard deviation, and *p* < 0.05 was considered statistically significant. For microbiota analysis, the ASV table was rarefied before diversity analysis. Alpha- and beta-diversity analyses were performed in QIIME2. Differential taxa were analyzed using LEfSe (http://huttenhower.sph.harvard.edu/galaxy/, accessed on 22 July 2026), and correlations between bacterial genera and urolithin profiles were evaluated using Spearman correlation analysis. The Benjamini–Hochberg procedure was used to control the false-discovery rate where applicable. Figures were prepared using OriginPro (2025b) and GraphPad Prism (10.4.1).

## 3. Results and Discussion

### 3.1. Visual Observation and Equilibrium Apparent Solubility Determination

**Figure 1:**
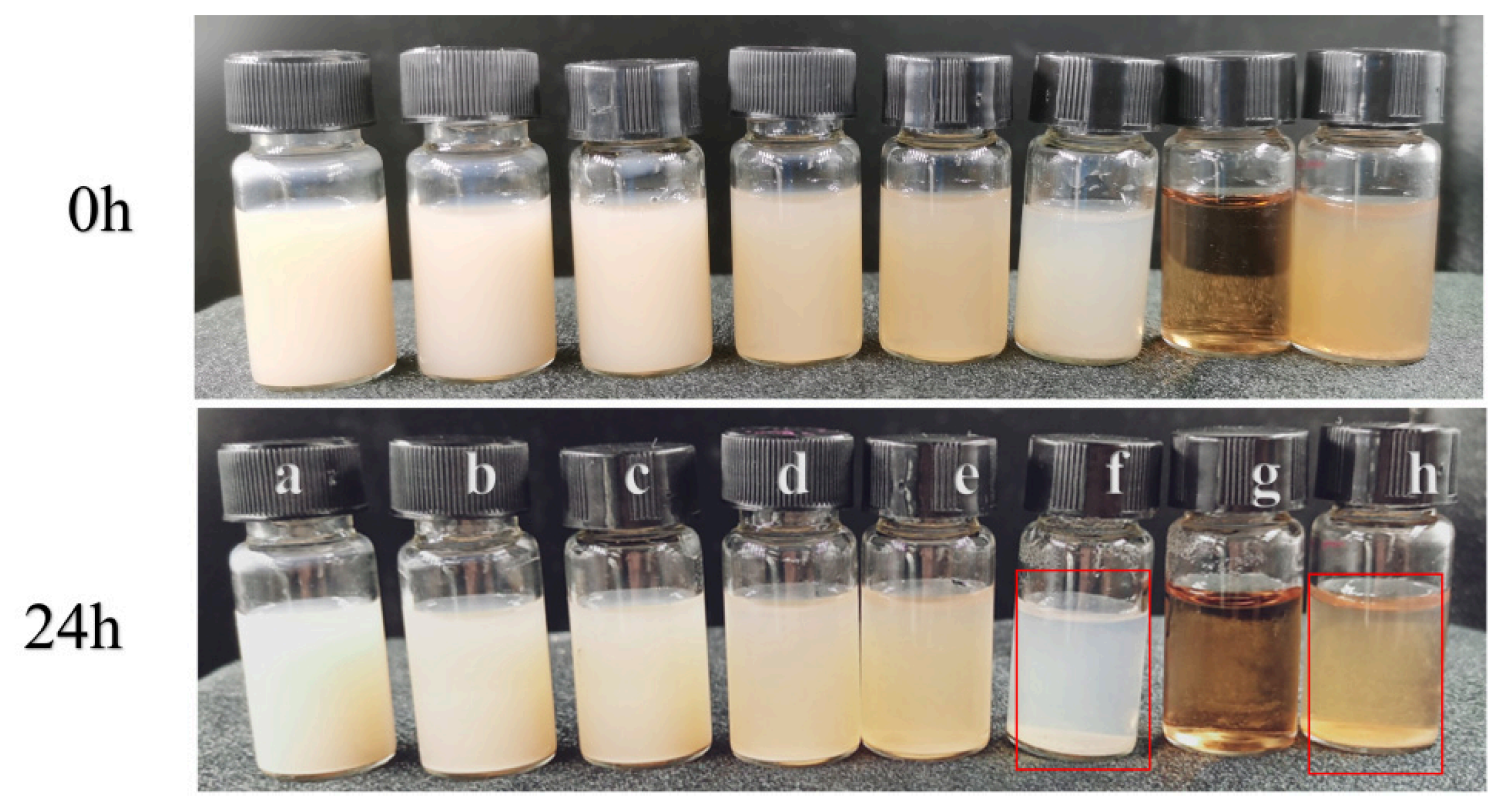
Visual observations of EA/FVP 5/1 (a), EA/FVP 3/1 (b), EA/FVP 1/1 (c), EA/FVP 1/3 (d), EA/FVP 1/5 (e), EA (f), FVP (g) and PM 1/1 (h) at 0 h and 24 h.

Figure 1 showed the visual appearances of the samples with the concentration of 0.1% (*w*/*v*) in aqueous solution at 0 h and 24 h. For the pure EA solution, only a small portion of EA dissolved, resulting in an opaque milky white solution with a large amount of sediment at the bottom. In contrast, FVP was completely soluble, forming a clear and nut-brown solution. EA/FVP SDs exhibited an opaque yellow-milky appearance, and the turbidity of the solutions increased with the rise in the EA mass ratio. The physical mixture of EA and FVP at an equal mass ratio (PM 1/1) was used as a control. Compared to PM 1/1 solution, the EA/FVP SDs solutions showed higher turbidity, indicating more effective dispersion of EA. After standing at room temperature for 24 h, large sediments were observed in both the pure EA and PM 1/1 solutions, and the supernatants became transparent. In contrast, the turbidity of the EA/FVP SDs solutions only slightly decreased, demonstrating that EA/FVP SDs could stably suspend EA in aqueous media.

**Figure 2:**
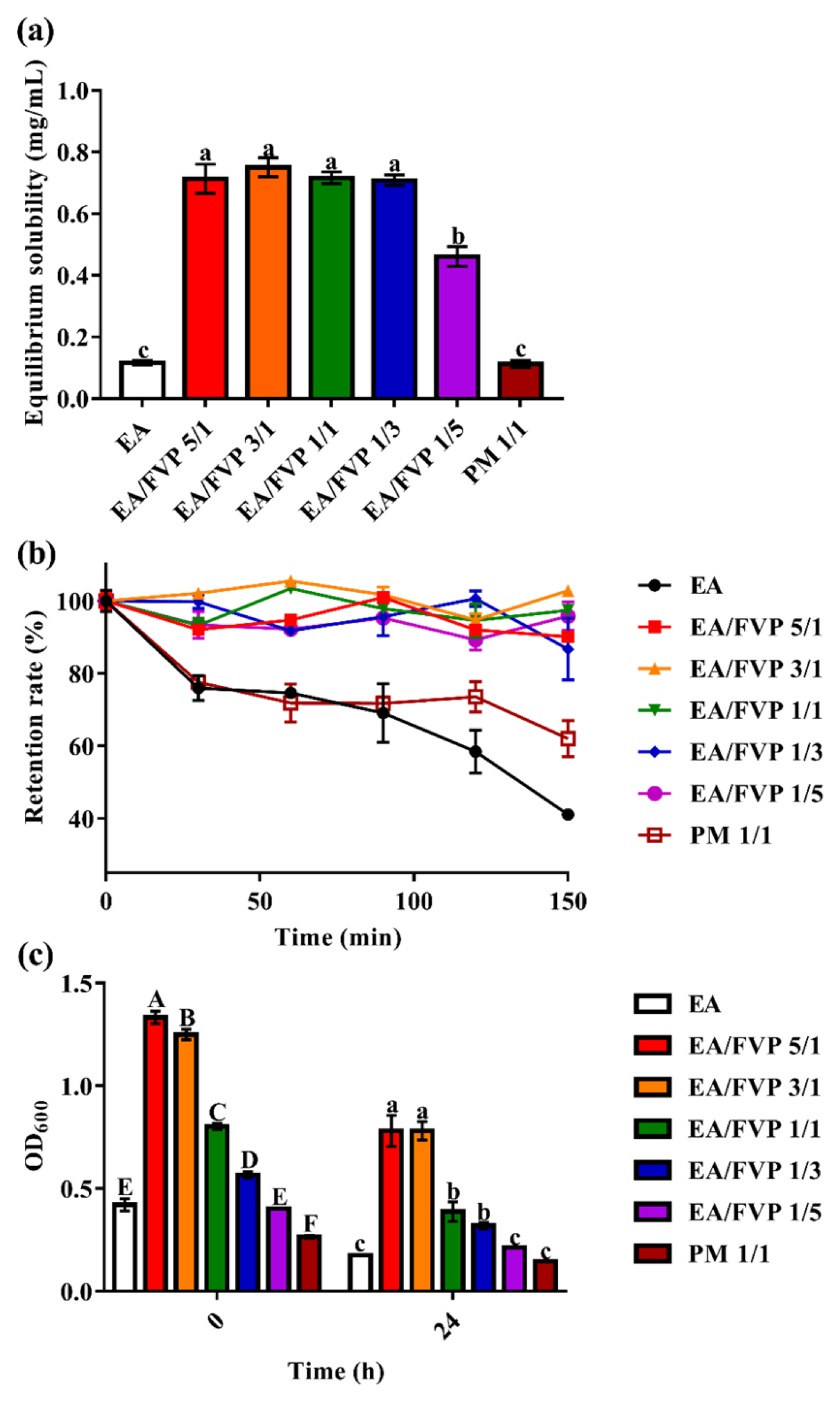
Equilibrium solubility (**a**), retention rate during 150 min (**b**) and the turbidity (**c**) of EA/FVP SDs. Data are expressed as mean ± SD (n = 3). Significant differences (*p* < 0.05) are indicated with different letters.

Figure 2a showed the equilibrium solubility of samples. The apparent equilibrium solubilities of EA in the pure EA solution and PM 1/1 solution were 0.12 ± 0.01 mg/mL and 0.11 ± 0.01 mg/mL, respectively, with no significant difference between the two groups. However, the equilibrium apparent solubilities of EA in the EA/FVP SDs solutions were significantly higher than that in the pure EA solution. Among the EA/FVP SDs with different mass ratios (5/1, 3/1, 1/1, and 1/3), there was no significant difference in EA apparent solubility, and all values exceeded 0.7 mg/mL. Notably, the EA/FVP 3/1 SDs exhibited the highest EA apparent solubility (0.75 ± 0.03 mg/mL). The improved apparent solubility may be associated with the ability of FVP to enhance aqueous dispersion, reduce EA aggregation, and maintain EA in a more stable suspended state. For the EA/FVP 1/5 SDs (with a relatively low EA/FVP ratio), the EA apparent solubility was 0.46 ± 0.03 mg/mL, which was significantly lower than that of the other EA/FVP SDs. This phenomenon can be explained by the fact that an excessively low EA/FVP ratio may hinder EA dissolution during the process; insufficient drug content combined with excess high-viscosity polymers can lead to the formation of fluffy flocculates in the solution. These flocculates cause EA accumulation at the interface, thereby reducing its dissolution efficiency [20]. Collectively, these results showed that EA/FVP SDs significantly improved the apparent solubility and aqueous dispersion of EA.

### 3.2. Stability Determination

An ideal SD is defined as a system where EA is uniformly dispersed under relatively stable suspension conditions [21]. Figure 2b,c showed the dispersity and short-term stability of samples at a concentration of 0.1% (*w*/*v*). As shown in Figure 2b, pure EA rapidly sedimented and the OD_600_ value dramatically decreased within 150 min. PM 1/1 showed a slowdown in the sedimentation of EA, indicating the addition of macromolecules would inhibit the aggregation of EA, but the retention rate was only 61.98% after 150 min. In contrast, EA/FVP SDs expressed superior short-term stability of EA, with the retention rate fluctuating around 90% at 150 min. A similar tendency was observed in Figure 2c. At the beginning, the OD_600_ values of EA/FVP SDs significantly increased with the elevation of the EA mass ratio, suggesting that EA/FVP SDs can effectively disperse EA in aqueous solutions. After 24 h, there was no significant difference in the OD_600_ values among EA, EA/FVP 1/5 and PM 1/1. The result was consistent with the visual observation in Figure 1. Notably, the EA/FVP 3/1 SDs showed the best performance in maintaining a stably dispersed EA suspension within 24 h. Collectively, these findings demonstrated that EA/FVP SDs could significantly enhance the dispersibility and stability of EA in aqueous solutions.

### 3.3. Size and Zeta Potential

Particle size and zeta potential are important physical characteristics for evaluating the stability of a suspension [22]. Table 1 summarizes the particle size, polydispersity index (PDI), and zeta potential of the samples at a concentration of 0.02% (*w*/*v*). The results showed that the average particle size of pure EA was greater than 1000 nm with a PDI value of 0.419 ± 0.195. This suggested that the insoluble EA particles tend to aggregate and form precipitation in aqueous solution. For *Fucus vesiculosus* polysaccharide (FVP) alone, despite its long-chain molecular structure resulting in an average particle size greater than 1000 nm, it maintained a stable soluble state in the solution. A simple physical mixture of FVP and EA would not decrease the particle size. In contrast, EA/FVP SDs showed a significant improvement in the unstable aggregation state of EA; both the average particle size and PDI values of the SDs decreased remarkably compared to pure EA and PM 1/1. All PDI values of the EA/FVP SDs were below 0.30, indicating that the particles in these solutions had uniform size distributions. Additionally, with the decrease in the EA/FVP mass ratio, the average particle size of the SDs first decreased and then increased. Among all SD formulations, the EA/FVP 1/1 SD exhibited the smallest average particle size, reaching 538.48 ± 16.52 nm.

Zeta potential determination measured the particle surface charge in the solution, and indicated the stability of the solution on a macroscopic scale. A well-accepted criterion for colloidal stability is that a system can maintain stability when the absolute value of zeta potential exceeds 30 mV. As shown in Table 1, the absolute values of all samples except EA exceeded 40 mV, indicating good colloidal stability. As the FVP ratio increased in EA/FVP SDs, the potential greatly decreased, suggesting strong interactions between FVP and EA occurred. Among all formulations, the EA/FVP 1/1 exhibited the smallest average particle size and a high zeta potential value. Collectively, these results demonstrated that EA/FVP SDs enhanced the apparent solubility of EA through multiple mechanisms: significantly reducing the particle size of EA, inhibiting EA particle aggregation, and improving the colloidal stability of the aqueous suspension system. These combined effects ultimately promoted the dissolution and dispersion of poorly soluble EA in water.

### 3.4. Ultraviolet–Visible Spectroscopy Analysis

**Figure 3:**
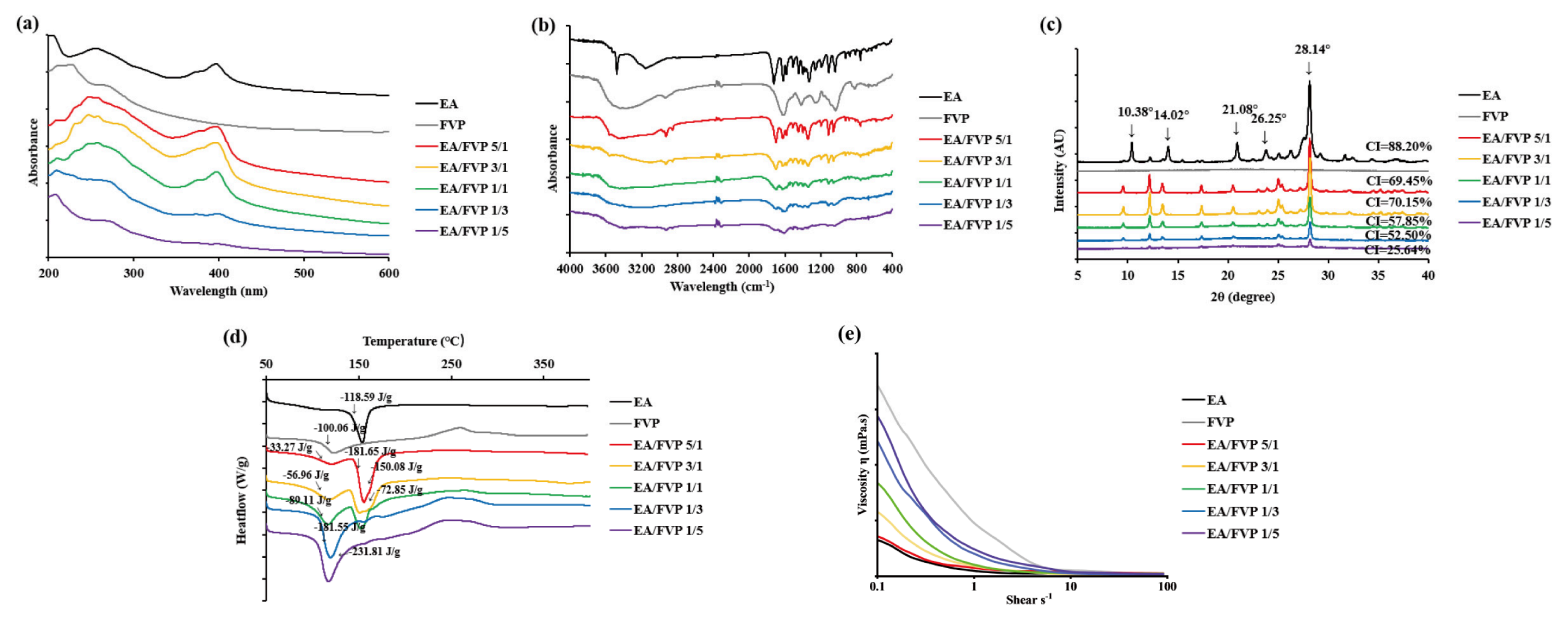
UV–visible spectroscopy (**a**), FT-IR spectra (**b**), X-ray diffraction patterns (**c**), DSC curves (**d**) and viscosity curves (**e**).

The ultraviolet–visible spectroscopy was employed to investigate the optical properties of samples. As apparent from Figure 3a, pure EA exhibited characteristic absorption peaks at 255 nm and 397 nm, indicating the presence of benzene ring and lactone ring in its structure. FVP showed absorption peak at 226 nm, which might be related to the sulfuric acid group in the algal polysaccharides. In EA/FVP SDs, a hyperchromic effect was observed as the proportion of EA increased. It was attributed to the enhanced suspension stability of EA in the SDs, which allowed more EA molecules to be detected in the aqueous system. A similar trend was reported in a previous study, where samples with higher EA concentrations exhibited stronger absorption intensities [23]. Furthermore, shifts in the red absorption peaks were observed in EA/FVP 5/1, EA/FVP 3/1 and EA/FVP 1/1. The increased absorption intensity of EA in the SDs was consistent with the previously observed enhancements in solubility and dispersibility, further confirming the effective dispersion of EA by FVP in the solid dispersion system.

### 3.5. FT-IR Spectra

When characterizing the molecular structures, infrared spectroscopy is often used to identify and determine the chemical functional groups of compounds and investigate intermolecular interactions during system formation. In Figure 3b, pure EA exhibited characteristic absorption peaks at 3473 cm^−1^, 1330 cm^−1^ and 1726 cm^−1^, which correspond to the stretching vibrations of -OH, phenol-OH and C-O groups, respectively. For FVP, the bands with absorption peaks of 3142 cm^−1^, 1622 cm^−1^, and 1302 cm^−1^ correspond to the stretching vibrations of the -OH group, and the stretching vibration of the asymmetric and symmetric-COO- groups, which matched previously reported peaks [24]. As expected, the characteristic peaks of both EA and FVP were broadened in the FT-IR spectra of the EA/FVP SDs, indicating that EA was successfully encapsulated in the FVP matrix. Specifically, the -OH stretching peak of EA at 3473 cm^−1^ shifted to lower wavenumbers (3360–3420 cm^−1^) and was markedly broadened, forming a broad absorption envelope in the range of 3360–2840 cm^−1^, which was attributed to the stretching vibration of phenolic O-H groups involved in intermolecular interactions. These spectral changes suggest the formation of hydrogen bonds or other non-covalent interactions between FVP and EA, contributing to the stable dispersion of EA in the SD system. The FT-IR spectra of the EA/FVP SDs retained almost all characteristic peaks of EA and FVP without the emergence of new absorption peaks. This result confirmed that the chemical structure of EA remained unchanged after the formation of the solid dispersion, indicating that the interaction between EA and FVP was physical rather than chemical.

### 3.6. XRD Analysis

The X-ray patterns of EA, FVP and EA/FVP SDs are shown in Figure 3c. There were several obvious peaks at the diffraction angles (2*θ*) of 10.38°, 14.02°, 21.08°, 26.25° and 28.14° in EA, confirming that EA possesses a typical crystalline structure [25]. According to Bragg’s law (lambda = 2dsin theta, lambda = 0.15418 nm for Cu-Kalpha radiation), the corresponding d-spacing values were calculated as 0.852, 0.631, 0.421, 0.339 and 0.317 nm, respectively. FVP was an amorphous macromolecule without obvious crystalline peaks. It is well known that solid dispersions could convert crystalline compounds into amorphous forms, thereby improving their solubility and dissolution rate [26]. FVP was an amorphous macromolecule without obvious peaks in the X-ray pattern, thus it could be an ideal material. After forming the EA/FVP SDs, the characteristic diffraction peaks of EA were significantly weakened, and the crystallinity index (CI) decreased from 88.20% to 25.64%. The reduction in the crystallinity was presumably attributed to the formation of hydrogen bonding between EA and FVP, which disrupts the ordered crystal lattice of EA. According to a previous study, the decrease in the degree of crystallization was the main factor for the increase in solubility [27]. However, the equilibrium solubility tendency of EA/FVP SDs did not fully align with their crystallinity degree. This inconsistency suggested that other structural factors (e.g., intermolecular interactions, particle size reduction, or dispersion state) also played important roles in enhancing the solubility of EA.

### 3.7. DSC Analysis

DSC is a thermal analysis method that measures temperature characteristic points such as phase transition temperature. The areas of exothermic peaks and absorption peaks in the curve can reflect the heat released or absorbed during phase transition. In the current study, Figure 3d exhibits the thermal stability of EA/FVP SDs using DSC. Pure EA showed an endothermic melting peak around 154 °C with a ΔH value of −118.59 J/g, while FVP presented an endothermic melting peak at 123 °C with a ΔH value of −100.06 J/g. No distinct glass transition temperature was observed for the EA/FVP SDs under the tested conditions, indicating that the system did not form a single homogeneous glassy phase. In the DSC curves of EA/FVP SDs, two distinct endothermic signals (corresponding to EA and FVP) were still detectable. With the increase in the EA/FVP mass ratio, the endothermic peak of FVP gradually weakened and shifted to a lower temperature, while the endothermic peak of EA became increasingly prominent. Specifically, the endothermic peak at 154 °C disappeared into the curves of EA/FVP 1/3 and EA/FVP 1/5. This observation indicated a reduction in EA crystallinity, which was consistent with the results of XRD analysis. These results suggest that EA exists in a less crystalline or highly dispersed state within the FVP matrix, and the dispersion structure can remain stable under the current preparation and drying temperature conditions. Furthermore, the hydrogen bonds between EA and FVP were presumably the main intermolecular force maintaining the tertiary structure of the SD system. However, long-term thermal stability and storage stability under different humidity, temperature and light conditions still require further systematic evaluation.

### 3.8. Steady-State Shear Rheological Behavior

**Figure 4:**
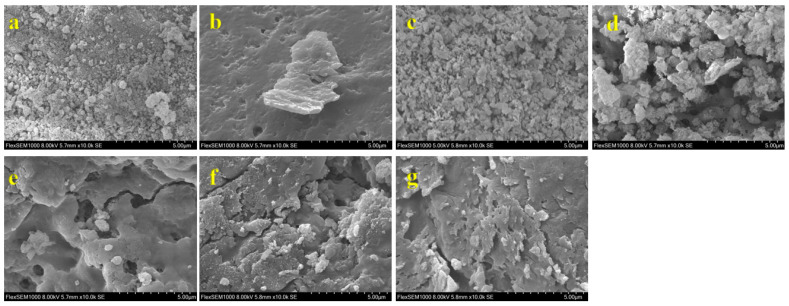
SEM images of EA (**a**), FVP (**b**), EA/FVP 5/1 (**c**), EA/FVP 3/1 (**d**), EA/FVP 1/1 (**e**), EA/FVP 1/3 (**f**) and EA/FVP 1/5 (**g**).

Steady-state shear rheological analysis was conducted to further evaluate the contribution of FVP to the dispersion behavior of EA/FVP SDs. As shown in Figure 4e, FVP and all FVP-containing systems exhibited typical shear-thinning behavior, with apparent viscosity decreasing continuously as the shear rate increased from 0.1 to 100 s^−1^. Pure EA showed the lowest apparent viscosity over the tested shear rate range, whereas pure FVP exhibited the highest value. Among the EA/FVP SDs, the apparent viscosity rose gradually with increasing FVP proportion, and this difference was more prominent in the low-shear region. These results indicated that FVP increased the viscosity of the aqueous dispersion and contributed to maintaining EA in a stable suspended state, which was consistent with the improved short-term suspension stability observed in the turbidity assay.

### 3.9. SEM Analysis

SEM was employed to observe the surface morphology and microstructural characteristics of pure EA, FVP and EA/FVP SDs. As shown in Figure 4a, pure EA exhibited a rough surface with plenty of spherical, micron-sized, clustered particles. Conversely, FVP exhibited a uniform sheet-like morphology with a relatively smooth surface (Figure 4b). Figure 4c–g shows the appearances of EA/FVP SDs. The pictures showed that the agglomerated EA particles were uniformly adsorbed and dispersed on the surface of FVP. In EA/FVP SDs, FVP served as a supporting matrix for EA dispersion, and this dispersed morphology increased the contact area between EA and the aqueous medium, which was conducive to improving its dissolution efficiency [27]. With the proportion of EA increased, the surface became rougher with micropores. These pores were presumably generated by the volatilization of ethanol during the co-precipitation preparation of SDs, and the structure could affect the solubility and dissolution of EA [28]. Among the EA/FVP SD formulations, EA/FVP 3/1 showed the most uniform EA dispersion and the least visible crystalline aggregation, which was consistent with the results in Figure 2. This observation further confirmed that the EA/FVP 3/1 SD possessed the optimal microstructure for enhancing EA dissolution.

### 3.10. In Vitro Fecal Fermentation

Based on the physicochemical characterization, EA/FVP 3/1, 1/1 and 1/3, which showed more favorable apparent solubility, aqueous dispersion, suspension stability and morphology, were selected as representative formulations for the subsequent in vitro fecal fermentation experiment.

Given the extremely low bioavailability of EA, its microbial metabolites urolithins are considered as a key pathway for exerting significant in vivo biological activity [29]. However, urolithins production is greatly dependent on the composition of gut microbiome. Based on the type of urolithins produced after EA intake, people are classified into three different urolithin metabotypes. Here, in vitro fecal fermentation combined with urolithin content determination was conducted to study the biotransformation of EA. In this study, after six days of fermentation with fecal bacterial suspension, urolithin M5, urolithin M6, urolithin C, iso-urolithin A and urolithin B were detected in the fermentation broths. This result indicates that the recruited volunteers belonged to urolithin metabotype B [30].

**Figure 5:**
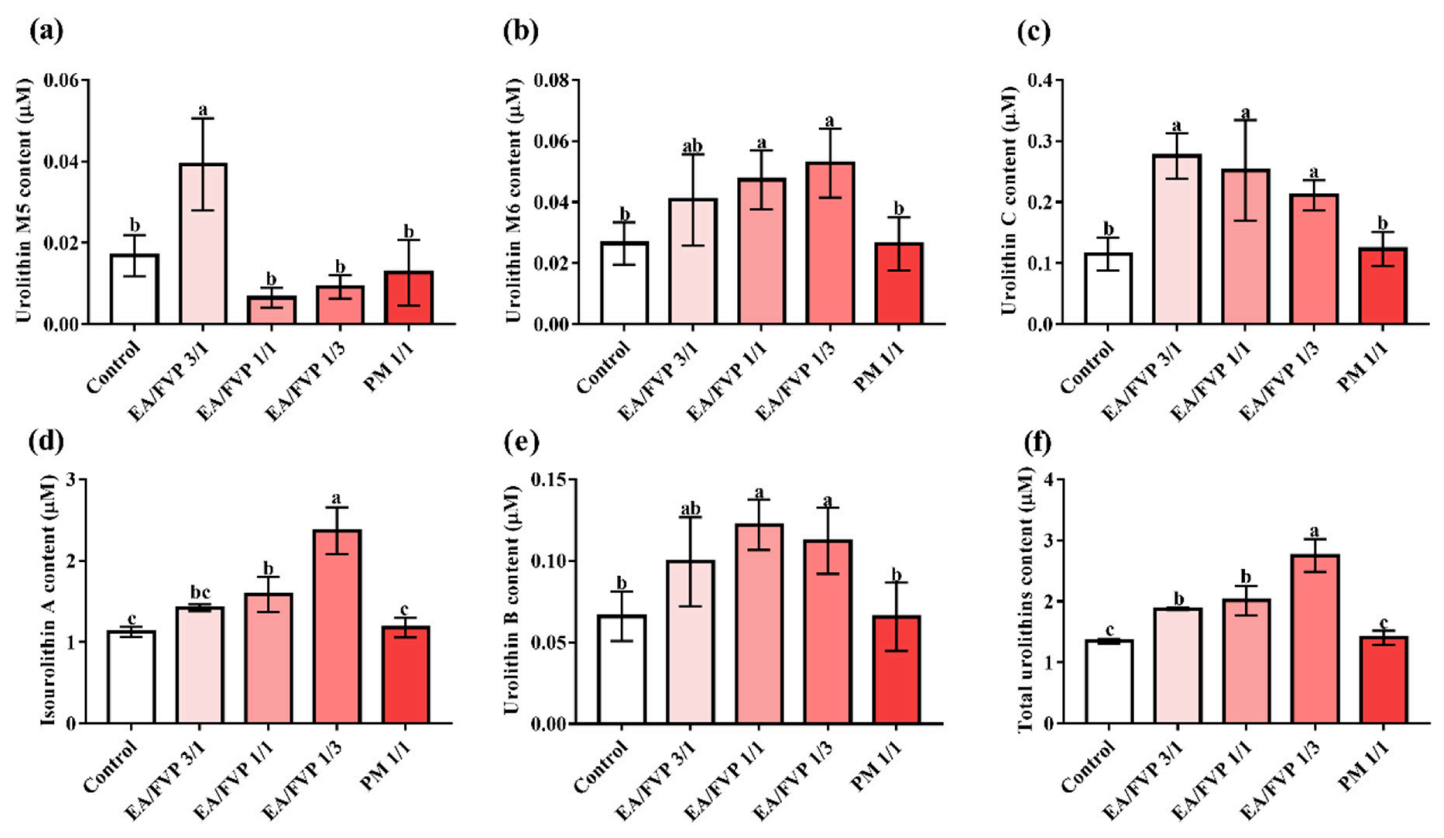
Contents of urolithin M5 (**a**), urolithin M6 (**b**), urolithin C (**c**), iso-urolithin A (**d**), urolithin B (**e**) and total urolithins (**f**) during fermentation among groups. Data are expressed as mean ± SD (n = 3). Significant differences (*p* < 0.05) are indicated with different letters.

As shown in Figure 5, there was no obvious differences noticed in the urolithin contents between the Control group and PM 1/1 group, indicating that the addition of FVP to the media could not directly improve the biotransformation process of EA. In other words, although FVP acts as a prebiotic, its proliferation-promoting effect targets gut bacteria that are not involved in urolithin biosynthesis. In contrast, the EA/FVP SDs significantly increased the urolithins contents but did not produce new urolithin types in broths. The total urolithins contents of the EA/FVP 3/1, EA/FVP 1/1 and EA/FVP 1/3 group were all notedly higher than those of the Control group and PM 1/1 group. Among the detected metabolites, the content of iso-urolithin A was the highest. The content of iso-urolithin A in the fermentation broth of EA/FVP 1/3 group was 2.36 ± 0.28 μM, which was significantly higher than that of other groups. Notably, although EA/FVP 3/1 showed the highest apparent solubility, EA/FVP 1/3 exhibited the strongest urolithin production capacity. This discrepancy suggests that microbial biotransformation is not determined by apparent solubility alone; particle size, crystallinity, dispersion stability, system viscosity and the higher FVP proportion may jointly affect EA accessibility and microbial metabolism during the longer fermentation period. The types of urolithins produced largely depended on the microflora compositions. Previous studies concluded that several bacteria could transform EA into some urolithins [31,32], but the metabolic pathway when inoculated with specific strains was incomplete compared to fecal bacteria fermentation, suggesting that other EA-metabolic-related strains and the interactions among microflora were still unknown. The current results showed that EA/FVP SDs enhance the bioconversion of EA by increasing its dispersibility and microbial accessibility, alongside the potential microbiota-modulatory effect of the FVP matrix.

### 3.11. Gut Bacteria Analysis

**Figure 6:**
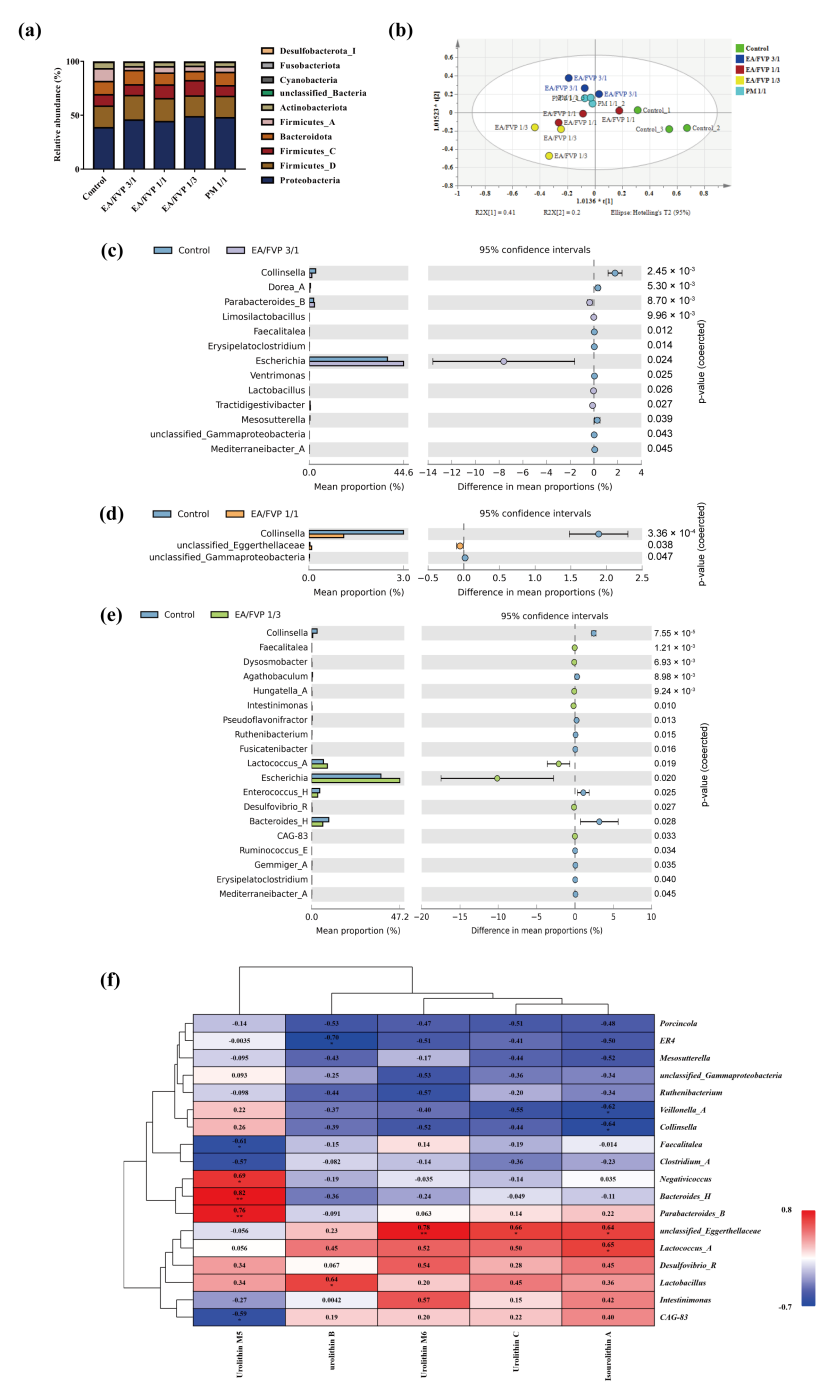
Relative abundance of phylum in broths (**a**), OPLS-DA plot (**b**), LEfSe analysis (**c**–**e**) and Spearman’s correlations between urolithin profiles and gut microflora (* *p* < 0.1, ** *p* < 0.01) (**f**). The differences between groups were determined using Welch’s *t*-test, and the Benjamini–Hochberg procedure was used to control the false-discovery rate due to multiple testing.

In this study, 16S rRNA sequencing analysis was performed to investigate the potential impacts of EA/FVP SDs supplements on the gut microflora. Figure 6a displayed the relative abundance of bacterial compositions at the phylum level. Compared to the Control group, the relative abundances of *Desulfobacterota*_I and *Proteobacteria* increased in EA/FVP SDs groups, while the relative abundances of *Actinobacteriota*, *Firmicutes*_A and unclassified_*Bacteria* decreased. These changes suggest that EA/FVP SDs may be associated with shifts in bacterial groups related to EA metabolism during in vitro fermentation. The whole composition of the bacteria was reflected using an OPLS-DA plot in Figure 6b. Separations among each group were noticed in the community structures, indicating that the treatments affected the microbial community profile. In particular, the distance between the EA/FVP 1/3 group and Control group was the farthest, and the result was correlated with the urolithin production. This result suggested that EA/FVP 1/3 may alter the microbial community associated with EA metabolism. Furthermore, taxon-based analysis displayed apparent changes (*p* < 0.05) of the gut bacteria at the genus level in Figure 6c–e. EA/FVP 3/1 supplement notedly increased the relative abundances of *Escherichia*, *Parabacteroides*_B, *Lactobacillus* and *Tractidigestivibacter*, EA/FVP 1/1 elevated the relative abundance of unclassified_*Eggerthellaceae*, and EA/FVP 1/3 increased the relative abundances of *Escherichia*, *Lactococcus*_A, *Desulfovibrio*_R, *Dysosmobacter*, *Hungatella*_A and *Intestinimonas* as compared to the Control group. These results suggested that the above bacteria may be associated with urolithin production.

Figure 6f exhibits Spearman’s correlations between bacteria and the urolithins. The results showed that urolithin M5 content was positively correlated with the relative abundances of *Bacteroides*_H, *Negativicoccus* and *Parabacteroides*_B, and negatively correlated with the relative abundances of *Faecalitalea*, CAG-83 and *Clostridium*_A, and *Unclassified*_*Eggerthellaceae* and *Lactococcus*_A showed positive accordance with urolithin M6, urolithin C and isourolithin A, suggesting that these bacteria may be associated with EA biotransformation under the present in vitro fermentation conditions. Previous studies showed that *Gordonibacter pamelaeae* and *Gordonibacter urolithinfaciens* are involved in the metabolism of EA to produce urolithin C [33], and *Ellagibacter isourolithinifaciens* could produce isourolithin A [34]. Urolithin B content was positively correlated with the relative abundance of *Lactobacillus* and negatively correlated with the relative abundance of ER4. These results suggested that bacteria such as *Bacteroides*_H, *Negativicoccus*, *Parabacteroides*_B, *Clostridium*_A. *Unclassified*_*Eggerthellaceae* and *Lactococcus*_A may be associated with EA metabolism. In addition, the positive association between several opportunistic or potentially unfavorable taxa and urolithin production suggested that EA conversion may vary with the background microbial community structure, which requires further validation through targeted studies.

## 4. Conclusions

In this study, FVP was used as a natural carrier to improve the apparent solubility and aqueous dispersion of EA, thereby facilitating its in vitro microbial biotransformation into urolithins. Consequently, EA/FVP SDs with different mass ratios were successfully prepared by solvent evaporation. Compared to the pure EA and simple physical mixture (PM 1/1), EA/FVP SDs exhibited better apparent solubility and stable suspension properties, which were mainly attributed to possible hydrogen bonding and other non-covalent interactions between EA and FVP. Comprehensive characterization results showed that EA/FVP SDs could inhibit the self-aggregation of EA, reduce its particle size and PDI, and decrease the crystallinity of EA, leading to a relatively stable maintenance of EA dispersion in aqueous solution. Additionally, rheological analysis revealed that FVP-containing systems exhibited shear-thinning behavior and increased apparent viscosity, which contributed to maintaining the stable dispersion of EA in aqueous solution. In vitro anaerobic fermentation showed that the EA/FVP SDs enhanced the biotransformation capacity of EA by improving its dispersibility, thereby facilitating the accessibility of EA to gut bacteria. Among the solid dispersions, EA/FVP 1/3 exhibited the best performance in improving urolithin productions. 16S rRNA sequence analysis indicated that the enhanced EA bioconversion was closely associated with shifts in gut microbial community composition. Specifically, EA/FVP 1/3 altered the relative abundances of *Escherichia*, *Lactococcus*_A, *Desulfovibrio*_R, *Dysosmobacter*, *Hungatella*_A and *Intestinimonas* in the broths. Furthermore, correlation analysis suggested that *Bacteroides*_H, *Negativicoccus*, *Parabacteroides*_B, *Unclassified_Eggerthellaceae* and *Lactococcus*_A, may be associated with urolithin production. These associations indicate potential links between microbial community shifts and EA bioconversion, but targeted studies are still needed to confirm their direct metabolic roles. The food-matrix stability, safety, bioavailability and in vivo validation of EA/FVP SDs require further investigation.

## Figures and Tables

**Table 1:** Particle size, PDI and zeta potentials of EA/FVP SDs. Data are expressed as mean ± SD (n = 3). Significant differences (*p* < 0.05) are indicated with different letters.

Group	Average Particle Size (nm)	PDI	Zeta Potential (mV)
EA	>1000 ^e^	0.419 ± 0.195 ^a^	−29.31 ± 1.14 ^a^
FVP	>1000 ^f^	0.271 ± 0.022 ^b^	−53.31 ± 1.47 ^d^
EA/FVP 5/1	791.42 ± 10.33 ^a^	0.269 ± 0.026 ^b^	−43.73 ± 3.35 ^b^
EA/FVP 3/1	680.69 ± 37.11 ^b^	0.296 ± 0.051 ^ab^	−51.84 ± 0.92 ^c^
EA/FVP 1/1	538.48 ± 16.52 ^d^	0.277 ± 0.010 ^b^	−58.35 ± 3.52 ^e^
EA/FVP 1/3	599.55 ± 9.90 ^c^	0.282 ± 0.024 ^b^	−68.25 ± 3.95 ^f^
EA/FVP 1/5	615.10 ± 34.80 ^c^	0.257 ± 0.050 ^b^	−68.27 ± 1.26 ^f^
PM 1/1	>1000 ^g^	0.302 ± 0.093 ^ab^	−57.51 ± 1.52 ^e^

## Data Availability

The original contributions presented in this study are included in the article. Further inquiries can be directed to the corresponding author.
